# ^68^Ga-PSMA-11 PET and mpMRI in the diagnosis of initial lymph node staging of prostate cancer: a head-to-head comparative meta-analysis

**DOI:** 10.3389/fmed.2024.1425134

**Published:** 2024-06-20

**Authors:** Yuanrong Wang, Ren Jing, Haiyan Wang, Qiuyan Zhao

**Affiliations:** ^1^Department of Geriatric Medical Center, West China Hospital of Sichuan University, Chengdu, China; ^2^Department of International Medical Center, West China Hospital of Sichuan University, Chengdu, China; ^3^Outpatient Department, West China Hospital of Sichuan University, Chengdu, China

**Keywords:** ^68^Ga-PSMA-PET, mpMRI, lymph node metastases, prostate cancer, meta-analysis

## Abstract

**Purpose:**

This meta-analysis evaluates the comparative diagnostic efficacy of ^68^Ga-prostate-specific membrane antigen-11 PET (^68^Ga-PSMA-11 PET) and multiparametric MRI (mpMRI) for the initial lymph node staging of prostate cancer.

**Methods:**

We searched PubMed and Embase databases through October 2023 for studies that provide a head-to-head comparison of ^68^Ga-PSMA-11 PET and mpMRI, using pelvic lymph node dissection as the gold standard. We assessed sensitivity and specificity using the DerSimonian and Laird method, with variance stabilization via the Freeman-Tukey double inverse sine transformation. The quality of included studies was evaluated using the Quality Assessment of Diagnostic Performance Studies (QUADAS-2) tool.

**Results:**

The meta-analysis incorporated 13 articles, involving a total of 1,527 patients. ^68^Ga-PSMA-11 PET demonstrated an overall sensitivity of 0.73 (95% CI: 0.51–0.91) and a specificity of 0.94 (95% CI: 0.88–0.99). In comparison, mpMRI showed a sensitivity of 0.49 (95% CI: 0.30–0.68) and a specificity of 0.94 (95% CI: 0.88–0.99). Although ^68^Ga-PSMA-11 PET appeared to be more sensitive than mpMRI, the differences in sensitivity (*p* = 0.11) and specificity (*p* = 0.47) were not statistically significant.

**Conclusion:**

Our findings indicated that ^68^Ga-PSMA-11 PET and mpMRI exhibit similar sensitivity and specificity in the diagnosis of initial lymph node staging of prostate cancer. However, given that most included studies were retrospective, further prospective studies with larger sample sizes are essential to validate these results.

**Systematic Review Registration:**

PROSPERO code is CRD42023495266.

## 1 Introduction

The 2020 World Cancer Report data indicates that prostate cancer ranks as the 6th most common malignancy in terms of incidence and the 9th in terms of mortality among males (1). In the realm of prostate cancer diagnostics and therapeutics, the evaluation for lymph node metastasis is imperative, given its critical role as a prognostic determinant (2). The identification of cancer metastasis to lymph nodes aids physicians in gauging the disease’s severity, devising more targeted treatment strategies, and estimating patient survival rates and quality of life, thereby underscoring its significance in comprehensive prostate cancer management (3). Given the limitations of pelvic lymph node dissection (PLND) in terms of increased risk of complications and longer hospital stays, there has been a focus on alternative approaches to improve diagnostic accuracy while minimizing adverse effects (3, 4). Conventional imaging techniques such as computed tomography (CT) and magnetic resonance imaging (MRI) have been widely used to assess pelvic lymph nodes. However, their effectiveness is hindered by limitations in sensitivity, specificity, and spatial resolution (5, 6).

In recent years, ^68^Ga-prostate-specific membrane antigen-11 PET (^68^Ga-PSMA-11 PET) and multiparametric MRI (mpMRI) have emerged as promising technologies in enhancing the accuracy of initial lymph node staging in prostate cancer (7). These methods have garnered attention for their improved diagnostic precision, yet debates and research continue regarding their relative efficacy, reliability, and accessibility. Several studies present conflicting views on the comparative efficacy of ^68^Ga-PSMA-11 PET and mpMRI in prostate cancer staging. While some studies highlight the superior sensitivity of ^68^Ga-PSMA-11 PET, others report comparable diagnostic performance between the two modalities (8–20).

In light of these discrepancies, this meta-analysis aims to systematically evaluate and amalgamate existing research concerning the diagnostic accuracy of ^68^Ga-PSMA-11 PET and mpMRI in the initial lymph node staging of prostate cancer. To ensure consistency and reduce variability between studies, only those investigations where both modalities were utilized in the same patient cohort have been included.

## 2 Method

The methodology adhered to the Preferred Reporting Items for a Systematic Review and Meta-analysis of Diagnostic Test Accuracy (PRISMA-DTA) guidelines, ensuring comprehensive and transparent reporting (21). Furthermore, the protocol for this meta-analysis has been registered with PROSPERO (CRD42023495266).

### 2.1 Search strategy

To gather relevant literature, an extensive search was conducted across databases including PubMed and Embase, covering publications up to October 2023. The search strategy incorporated key terms such as “Positron-Emission Tomography,” “Multiparametric Magnetic Resonance Imaging,” and “Prostatic Neoplasms,” ensuring a focused approach to identifying pertinent studies. For more detailed information regarding the search strategy, refer to Supplementary Table S1.

Additionally, the reference lists of all included studies were manually scrutinized, aiming to uncover any additional relevant articles that may have been missed during the initial database search.

### 2.2 Inclusion and exclusion criteria

For inclusion in this meta-analysis, studies had to meet specific criteria: population (P): patients undergoing pelvic lymph node staging before radical prostatectomy for prostate cancer; intervention (I): ^68^Ga-PSMA-11 PET imaging; comparison (C): mpMRI imaging; outcome (O): sensitivity and specificity of each imaging modality in staging pelvic lymph nodes; study design (S): retrospective or prospective design.

Exclusion criteria were applied to maintain study quality and relevance: (1) duplicated articles, abstracts without full texts, editorial comments, letters, case reports, reviews, meta-analyses, irrelevant titles and abstracts, (2) non-English full-text articles were excluded; (3) studies lacking complete or clear data necessary for calculating sensitivity or specificity of the studied imaging modality; (4) patients less than 10; (5) without employing histopathology confirmation from PLND as the reference standard.

The screening process involved two researchers independently evaluating titles and abstracts of retrieved articles. Subsequently, full-text versions of remaining articles were assessed to determine their eligibility for inclusion. Any disagreements between the researchers were resolved through consensus.

### 2.3 Quality assessment

Two researchers independently assessed the quality and clinical applicability of included studies using the Quality Assessment of Diagnostic Performance Studies (QUADAS-2) tool, which encompasses four critical domains: patient selection, index test, reference standard, and flow and timing. Within each domain, the risk of bias and concerns regarding clinical applicability were categorized as “high risk,” “low risk,” or “unclear risk.” This systematic approach ensured a rigorous evaluation of study quality and provided insights into the practical relevance and applicability of the findings in clinical settings.

### 2.4 Data extraction

Two researchers independently conducted data extraction from the included articles, covering a range of essential information. This included details about the author, publication year, and the specific imaging test employed in the study. Additionally, data encompassed various study features such as country, design, analysis methods, and duration. Patient characteristics, such as the number of patients or lesions, PSA levels, mean or median age, and Gleason scores, were also extracted. Technical aspects such as scanner modality, ligand dosage, and image analysis techniques were included as well. In instances where discrepancies arose, the researchers engaged in discussions until a consensus was achieved, ensuring the accuracy and reliability of the extracted data.

### 2.5 Statistical analysis

The DerSimonian and Laird method was employed to assess sensitivity and specificity, which were then transformed using the Freeman–Tukey double inverse sine transformation. To determine the precision of these values, the Jackson method was used to calculate confidence intervals.

Heterogeneity within and between groups was evaluated using the Cochrane *Q* and *I*^2^ statistics. Significantly differing heterogeneity (*p* < 0.10 or *I*^2^ > 50%) prompted further investigation through meta-regression and sensitivity analysis to identify potential sources.

Publication bias was assessed using both funnel plot analysis and Egger’s test, ensuring the examination of potential reporting biases. Statistical significance was set at *p* < 0.05 for all tests conducted. All statistical analyses were performed using R software version 4.3.2.

## 3 Results

### 3.1 Study selection

**Figure 1:**
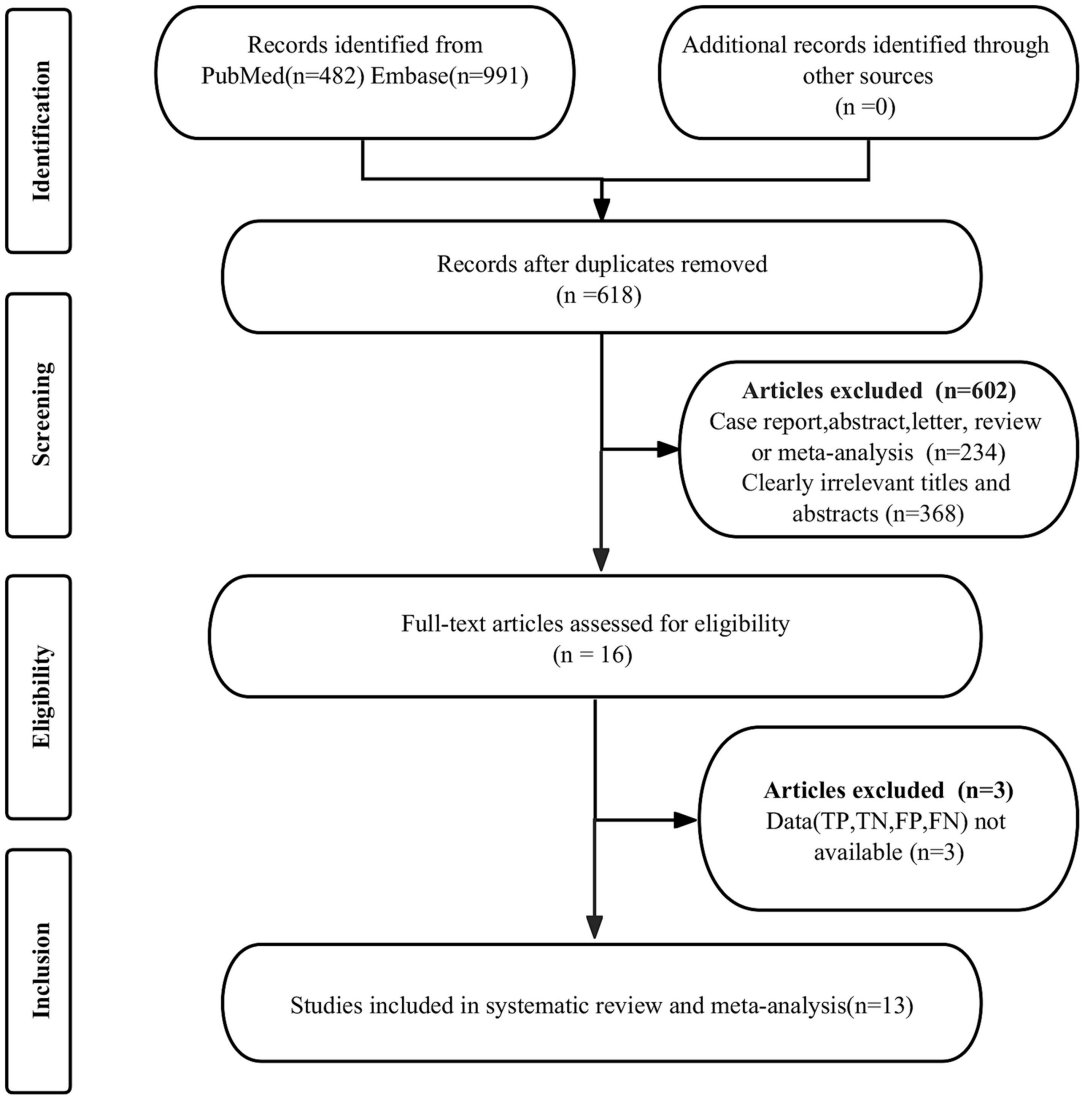
PRISMA flow diagram illustrating the study selection process.

The preliminary search identified 1,473 publications, of which 855 were duplicates and 602 did not meet the eligibility criteria, leading to their exclusion. Subsequently, a comprehensive review of the full texts of the remaining 16 articles was conducted. Among these, three articles were deemed ineligible due to missing data, resulting in their exclusion from the study. Ultimately, 13 articles that evaluated the diagnostic efficacy of ^68^Ga-PSMA-11 PET and mpMRI met all criteria and were included in the meta-analysis (8–20). The process of article selection of the PRISMA flow diagram was illustrated in Figure 1.

### 3.2 Study description and quality assessment

The 13 eligible studies encompassed a total of 1,527 breast cancer patients, with the sample size ranging from 10 to 780 across the studies. Among these, 12 articles adopted a retrospective study design, while 1 article employed a prospective approach. Regarding the analysis methods, 12 studies utilized patient-based analysis, while 1 study employed lesion-based analysis. Detailed characteristics of these studies are summarized in Tables 1, 2, providing an overview of the study and technique specifics related to ^68^Ga-PSMA-11 PET and mpMRI.

**Table 1:** Study and patient characteristics of the included studies.

Author	Year	Study characteristics				Number of patients (lesion)	Patient characteristics			
		Country	Study design	Analysis	Period		PSA level (ng/mL)	Age (year)	Gleason score	Reference standard
Hotker et al.	2023	Switzerland	Retro	PB	2016–2019	41	NA	Median: 65	Gleason = 7 (36.6%) Gleason ≥8 (63.4%)	PLND
Arslan et al.	2022	Turkey	Retro	LB	2015–2020	780	Median: 5.6	Mean: 62.47	Gleason = 7 (84.6%) Gleason ≥8 (15.4%)	ePLND
Skawran et al.	2022	Switzerland	Retro	PB	2016–2019	35	Median: 18.3	Mean: 66	Gleason = 7 (26.5%) Gleason ≥8 (73.5%)	PLND
Szigeti et al.	2022	Austria	Pro	PB	2017–2020	81	Mean: 15.4	Mean: 64.5	Gleason ≤6 (1.9%) Gleason = 7 (59.3%) Gleason ≥8 (38.8%)	ePLND
Damme et al.	2021	Belgium	Retro	PB	2016–2019	81	Median: 12.29	Median: 67	NA	PLND
Celen et al.	2020	Turkey	Retro	PB	NA	22	Mean: 9.49	Mean: 65.07	Gleason ≤6 (23.3%) Gleason = 7 (40.0%) Gleason ≥8 (36.7%)	PLND
Kulkarni et al.	2020	India	Retro	PB	2016–2018	51	Mean: 39.4	Mean: 66	Gleason ≤7 (51.0%) Gleason >7 (49.0%)	PLND
Frumer et al.	2020	Israel	Retro	PB	2016–2019	89	Median: 8.5	Median: 66.9	Gleason ≤7 (79.8%) Gleason >7 (20.2%)	PLND
Franklin et al.	2020	Australia	Retro	PB	2014–2019	233	Mean: 7.4	Mean: 68	NA	PLND
Yilmaz et al.	2019	Turkey	Retro	PB	2016–2018	10	NA	NA	NA	rPLND
Berger et al.	2018	Australia	Retro	PB	2015–2017	50	Mean: 10.6	Mean: 64.9	Gleason ≤6 (2%) Gleason = 7 (66%) Gleason ≥8 (32%)	ePLND
Gupta et al.	2017	India	Retro	PB	2014–2015	12	Median: 24.3	Mean: 61.75	Gleason ≤6 (8.3%) Gleason = 7 (16.7%) Gleason ≥8 (75%)	ePLND
Zhang et al.	2017	China	Retro	PB	2017	42	Mean: 52.31	Mean: 68.86	Gleason = 7 (42.9%) Gleason ≥8 (57.1%)	PLND

Pro, prospective; Retro, retrospective; PB, patient-based; LB, lesion-based; ePLND, extended lymph node dissection; rPLND, retroperitoneal lymph node dissection; PLND, lymph node dissection; NA, not available.

**Table 2:** Technical aspects of included studies.

Author	Year	Types of imaging tests	Scanner modality for PET	Scanner modality for mpMRI	Ligand dose	Image analysis	TP, FP, FN, TN (PET/CT)	TP, FP, FN, TN (mpMRI)
Hotker et al.	2023	[^68^Ga]Ga-PSMA PET vs. mpMRI	SIGNA PET/MR, GE Healthcare, Waukesha, United States; or Discovery MI PET/CT, GE Healthcare, Waukesha, WI, United States	MAGNETOM Skyra, Siemens Healthineers, Erlangen, Germany	2 MBq/kg	Visual and semiquantitative	TP: 7, FP: 0, FN: 4, TN: 30	TP: 9, FP: 15, FN: 2, TN: 15
Arslan et al.	2022	[^68^Ga]Ga-PSMA PET vs. mpMRI	GE Discovery 710 (General Electric, Milwaukee WI), GE Discovery IQ (General Electric, Milwaukee WI), or Siemens (Siemens, Erlangen, Germany) Biograph 20 mCT	3.0 T MR Siemens Healthineers, Magnetom Skyra, Erlangen, Germany	NA	Visual	TP: 2, FP: 4, FN: 9, TN: 765	TP: 4, FP: 3, FN: 7, TN: 766
Skawran et al.	2022	[^68^Ga]Ga-PSMA PET vs. mpMRI	SIGNA PET/MR; GE Healthcare, Waukesha, USA	MAGNETOM Skyra, Siemens Healthineers, Erlangen, Germany	134 ± 18.8 MBq	Visual and semiquantitative	TP: 5, FP: 0, FN: 4, TN: 26	TP: 9, FP: 3, FN: 5, TN: 6
Szigeti et al.	2022	[^68^Ga]Ga-PSMA PET vs. mpMRI	Philips Ingenuity TF, Amsterdam/the Netherlands, and Siemens Biograph mCT, Erlangen/Germany	Achieva, Philips Medical Systems, Best/The Netherlands	2.15 MBq/kg	Visual and semiquantitative	TP: 6, FP: 4, FN: 4, TN: 34	TP: 5, FP: 1, FN: 5, TN: 37
Damme et al.	2021	[^68^Ga]Ga-PSMA PET vs. mpMRI	PSMA-HBED-11 labelling kits provided by ABX, Germany; ^68^Ge/^68^Ga Galli Ad generator, IRE Elite, Fleurus, Belgium	Ingenia, Philips Medical Systems, The Netherlands	110 MBq	Visual and semiquantitative	TP: 31, FP: 2, FN: 0, TN: 48	TP: 18, FP: 0, FN: 9, TN: 54
Celen et al.	2020	[^68^Ga]Ga-PSMA PET vs. mpMRI	Gemini TF TOF PET-CT; Philips, Cleve-land, OH, United States	Ingenia, Philips Medical Systems, The Netherlands	125–317 MBq	Visual and semiquantitative	TP: 1, FP: 11, FN: 0, TN: 10	TP: 1, FP: 0, FN: 9, TN: 12
Kulkarni et al.	2020	[^68^Ga]Ga-PSMA PET vs. mpMRI	General Electric Medical Systems with eight slice helical CT scanner, Chicago, Illinois, United States	GE Discovery MR 750 W, Illinois, United States	111–166 MBq	Visual and semiquantitative	TP: 13, FP: 3, FN: 3, TN: 16	TP: 7, FP: 9, FN: 4, TN: 15
Frumer et al.	2020	[^68^Ga]Ga-PSMA PET vs. mpMRI	NA	3.0-T MR scanner or a 1.5-T scanner with a trans-rectal coil	112–187 MBq	Visual and semiquantitative	TP: 3, FP: 4, FN: 9, TN: 73	TP: 1, FP: 2, FN: 8, TN: 71
Franklin et al.	2020	[^68^Ga]Ga-PSMA PET vs. mpMRI	Philips Healthcare, Best, The Nederlands	Skyra; Siemens healthcare, Erlingen, Germany	200 MBq	Visual and semiquantitative	TP: 28, FP: 14, FN: 30, TN: 161	TP: 13, FP: 9, FN: 45, TN: 166
Yilmaz et al.	2019	[^68^Ga]Ga-PSMA PET vs. mpMRI	NA	Verio; Siemens Medical Solutions, Erlangen, Germany	175 MBq	Visual and semiquantitative	TP: 2, FP: 0, FN: 0, TN: 8	TP: 2, FP: 5, FN: 0, TN: 3
Berger et al.	2018	[^68^Ga]Ga-PSMA PET vs. mpMRI	Philips Gemini TF 64 PET/CT	3 Tesla machines	NA	Visual and semiquantitative	TP: 1, FP: 4, FN: 1, TN: 44	TP: 0, FP: 2, FN: 1, TN: 47
Gupta et al.	2017	[^68^Ga]Ga-PSMA PET vs. mpMRI	Biograph TruePoint40 with LSO crystal from Siemens Healthcare	NA	2 MBq/kg	Visual and semiquantitative	TP: 7, FP: 1, FN: 0, TN: 4	TP: 4, FP: 1, FN: 3, TN: 4
Zhang et al.	2017	[^68^Ga]Ga-PSMA PET vs. mpMRI	United Imaging Healthcare (UIH), Shanghai, China	Achieva 3.0 T TX, Philips Medical Systems, The Netherlands	130.6–177.6 MBq	Visual and semiquantitative	TP: 14, FP: 1, FN: 1, TN: 26	TP: 14, FP: 2, FN: 1, TN: 25

TP, true positive; TN, true negative; FP, false positive; FN, false positive; NA, not available.

**Figure 2:**
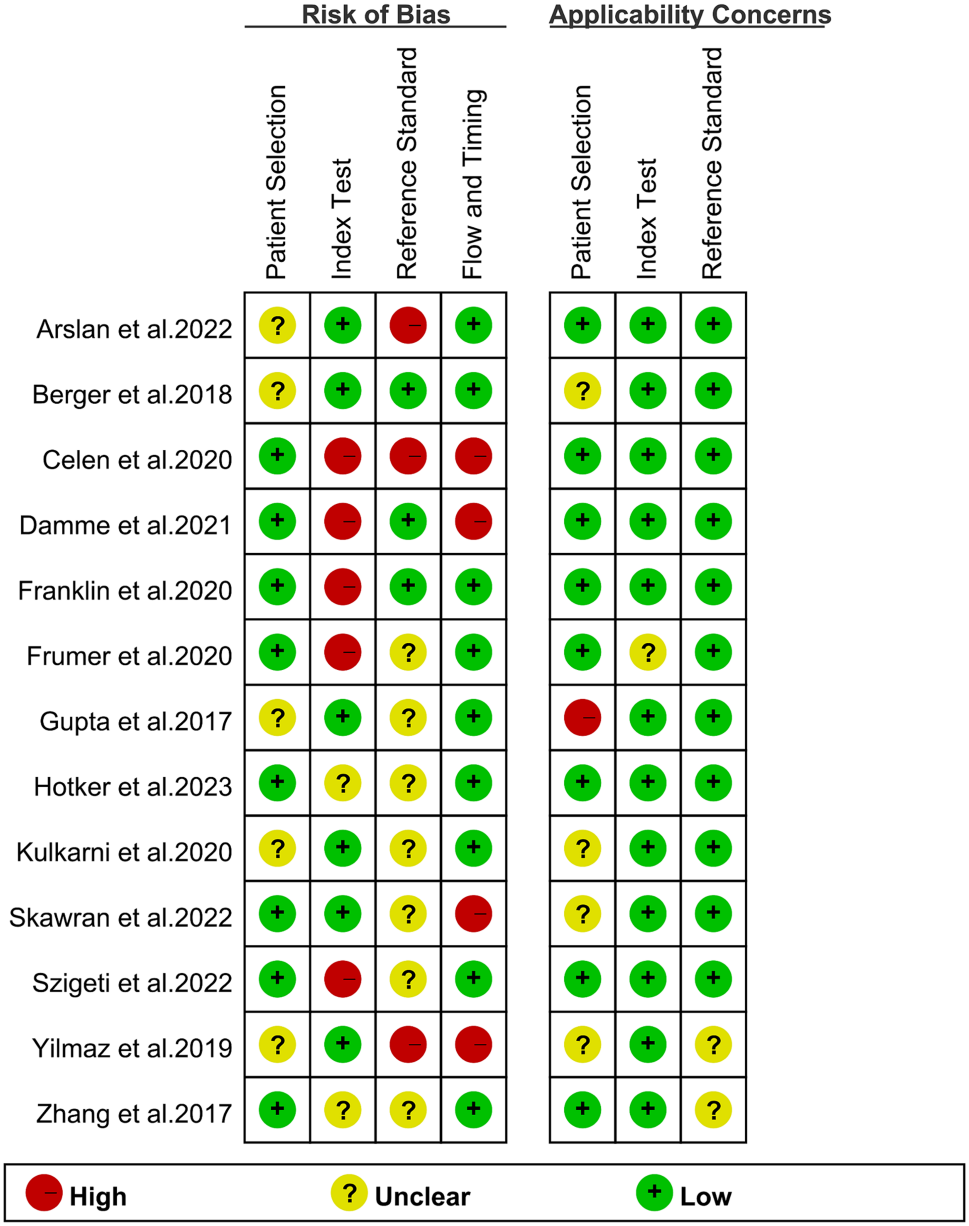
Risk of bias and applicability concerns of the included studies using the Quality Assessment of Diagnostic Performance Studies QUADAS-2 tool.

The risk of bias assessment, conducted using the QUADAS-2 tool, is visually represented in Figure 2. Specifically, 5 studies were identified as having a “high risk” in terms of the index test bias due to undetermined cut-off values. Additionally, 3 studies were categorized as “high risk” in terms of the reference standard bias as the final diagnosis lacked independent confirmation by multiple physicians. Moreover, 4 studies were graded as “high risk” in the flow and timing domain due to participant exclusion from data analyses. Overall, despite these specific biases identified, the overall quality assessment did not raise major concerns regarding the quality of the included studies.

### 3.3 Comparing the sensitivity of ^68^Ga-PSMA-11 PET and mpMRI for diagnosis of initial lymph node staging of prostate cancer

**Figure 3:**
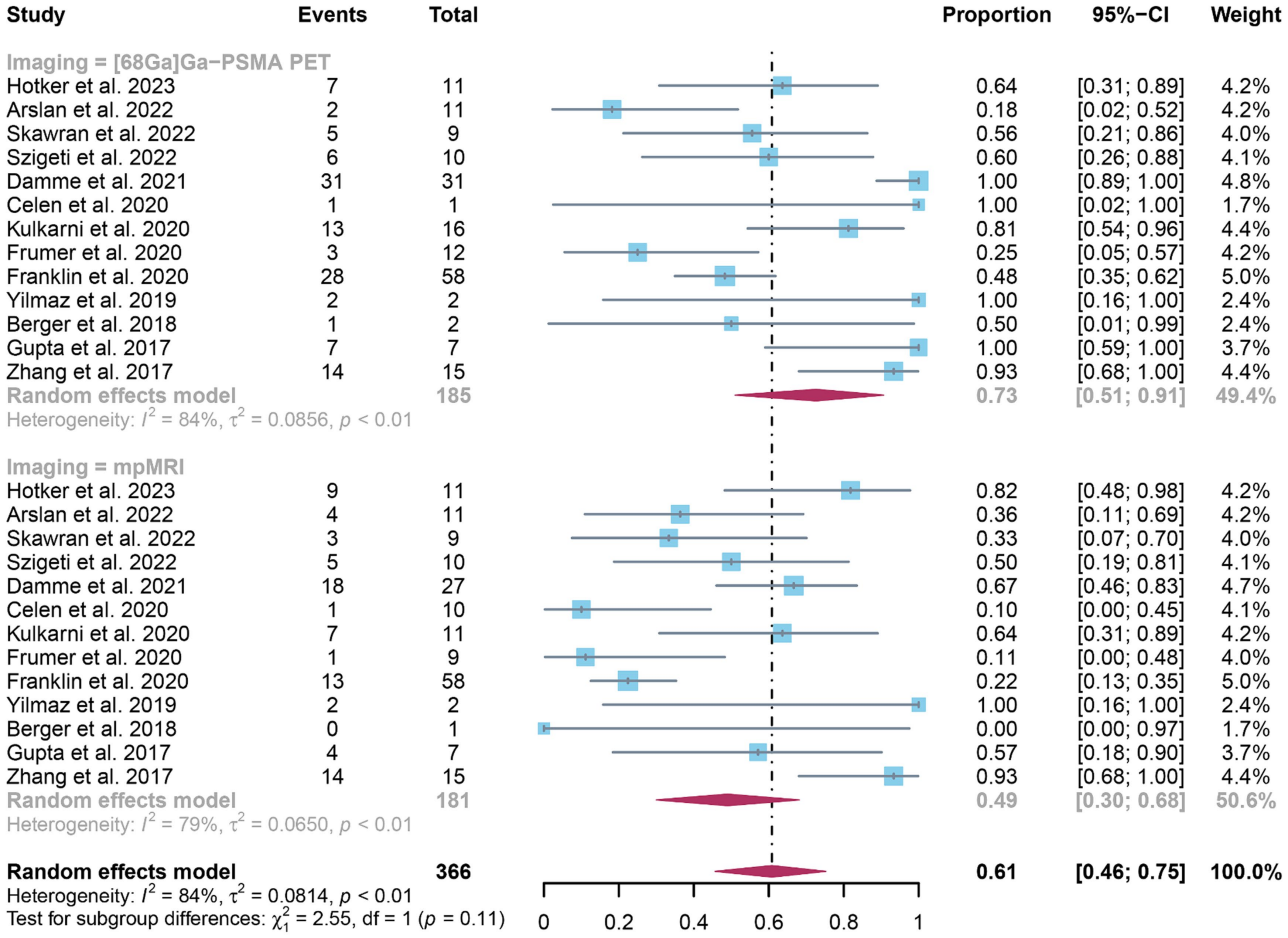
Forest plot showing the head-to-head comparison of pooled sensitivities of ^68^Ga-PSMA-11 PET and mpMRI in pelvic lymph node metastases of prostate cancer patients. The plot displays individual study estimates (squares) with corresponding 95% confidence intervals (horizontal lines) and the pooled sensitivity estimate (diamond) for both modalities. The size of the squares represents the relative weight of each study in the meta-analysis.

The analysis included a total of 13 studies. The pooled sensitivity of ^68^Ga-PSMA-11 PET for diagnosing initial lymph node staging of prostate cancer was 0.73 (95% CI: 0.51–0.91), while mpMRI showed an overall sensitivity of 0.49 (95% CI: 0.30–0.68), as depicted in Figure 3. Interestingly, there was no significant difference in sensitivity between ^68^Ga-PSMA-11 PET and mpMRI (*p* = 0.11), as indicated by Figure 3.

**Figure 4:**
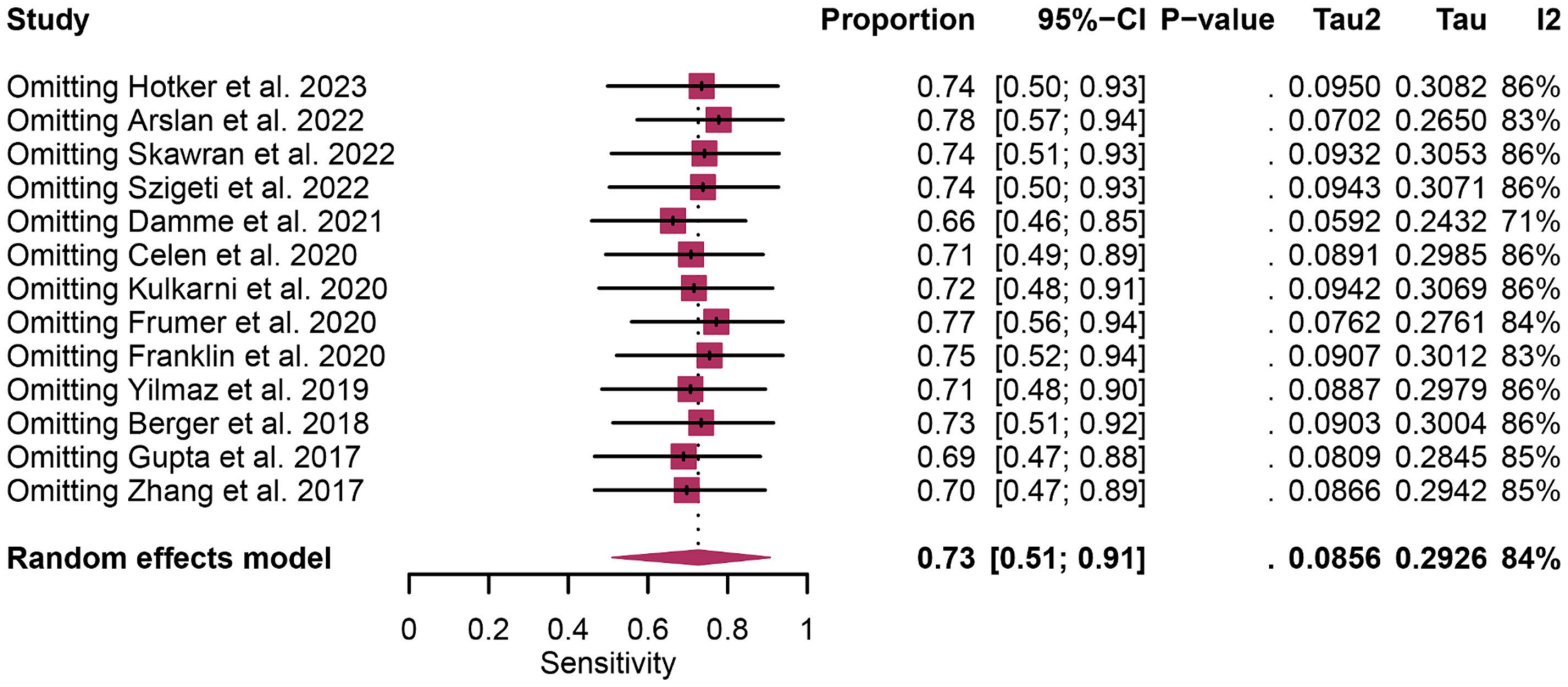
Forest plot showing the pooled sensitivity of ^68^Ga-PSMA-11 PET in pelvic lymph node metastases of prostate cancer patients. The plot displays individual study estimates (squares) with corresponding 95% confidence intervals (horizontal lines) and the pooled sensitivity estimate (diamond). The size of the squares represents the relative weight of each study in the meta-analysis.

**Figure 5:**
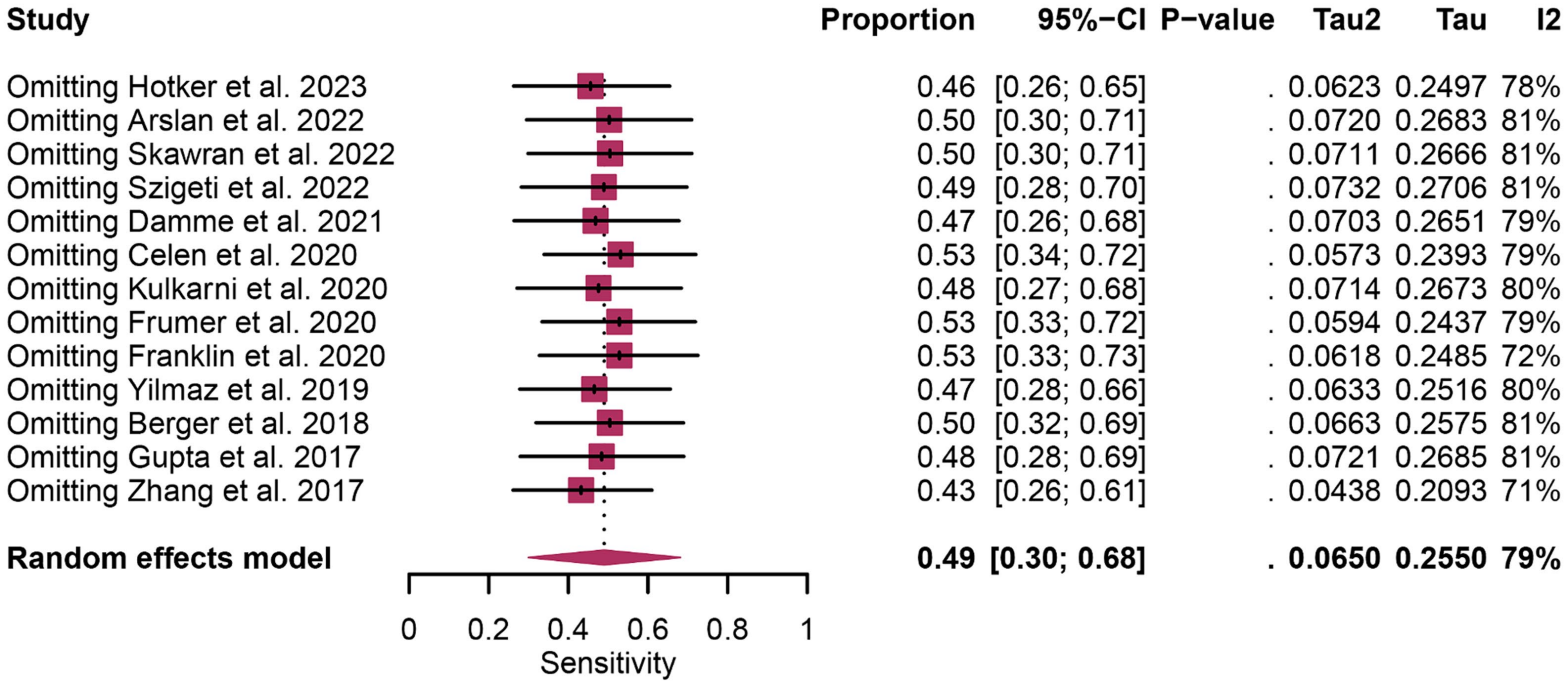
Forest plot showing the pooled sensitivity of mpMRI in pelvic lymph node metastases of prostate cancer patients. The plot displays individual study estimates (squares) with corresponding 95% confidence intervals (horizontal lines) and the pooled sensitivity estimate (diamond). The size of the squares represents the relative weight of each study in the meta-analysis.

In terms of heterogeneity, the *I*^2^ values for the pooled overall sensitivity were 84% for ^68^Ga-PSMA-11 PET and 79% for mpMRI, highlighting substantial heterogeneity (Figure 3). Despite this, meta-regression and sensitivity analysis did not identify any potential sources of heterogeneity, as illustrated in Figures 4, 5 and Table 3. Notably, the results from the sensitivity analysis remained stable, with only minor variations observed (ranging from 0.66 to 0.78 for ^68^Ga-PSMA-11 PET and from 0.43 to 0.53 for mpMRI), as shown in Figures 4, 5.

**Table 3:** Meta-regression analysis for sensitivity of ^68^Ga-PSMA-11 PET and mpMRI.

Covariate	Studies	Sensitivity for ^68^Ga-PSMA-11 PET (95% CI)	*p*-value	Sensitivity for mpMRI (95% CI)	*p*-value
Region			0.42		0.16
Oceania	2	0.48 [0.33; 0.63]		0.12 [0.02; 0.27]	
Asia	7	0.76 [0.40; 1.00]		0.51 [0.22; 0.80]	
Europe	4	0.77 [0.43; 0.99]		0.61 [0.42; 0.78]	
Number of patients included			0.33		0.35
>50	6	0.60 [0.29; 0.88]		0.41 [0.22; 0.61]	
≤50	7	0.87 [0.64; 1.00]		0.59 [0.25; 0.90]	
Reference standard			0.99		0.58
PLND	8	0.75 [0.49; 0.95]		0.48 [0.24; 0.72]	
ePLND	4	0.61 [0.16; 0.98]		0.43 [0.22; 0.66]	
rPLND	1	1.00 [0.16; 1.00]		1.00 [0.16; 1.00]	
Image analysis			0.11		0.69
Visual and semiquantitative	12	0.78 [0.57; 0.94]		0.50 [0.30; 0.71]	
Visual	1	0.18 [0.02; 0.52]		0.36 [0.11; 0.69]	
Study design			0.77		0.97
Prospective	1	0.60 [0.26; 0.88]		0.50 [0.19; 0.81]	
Retrospective	12	0.74 [0.50; 0.93]		0.49 [0.28; 0.70]	

### 3.4 Comparing the specificity of ^68^Ga-PSMA-11 PET and mpMRI for diagnosis of initial lymph node staging of prostate cancer

**Figure 6:**
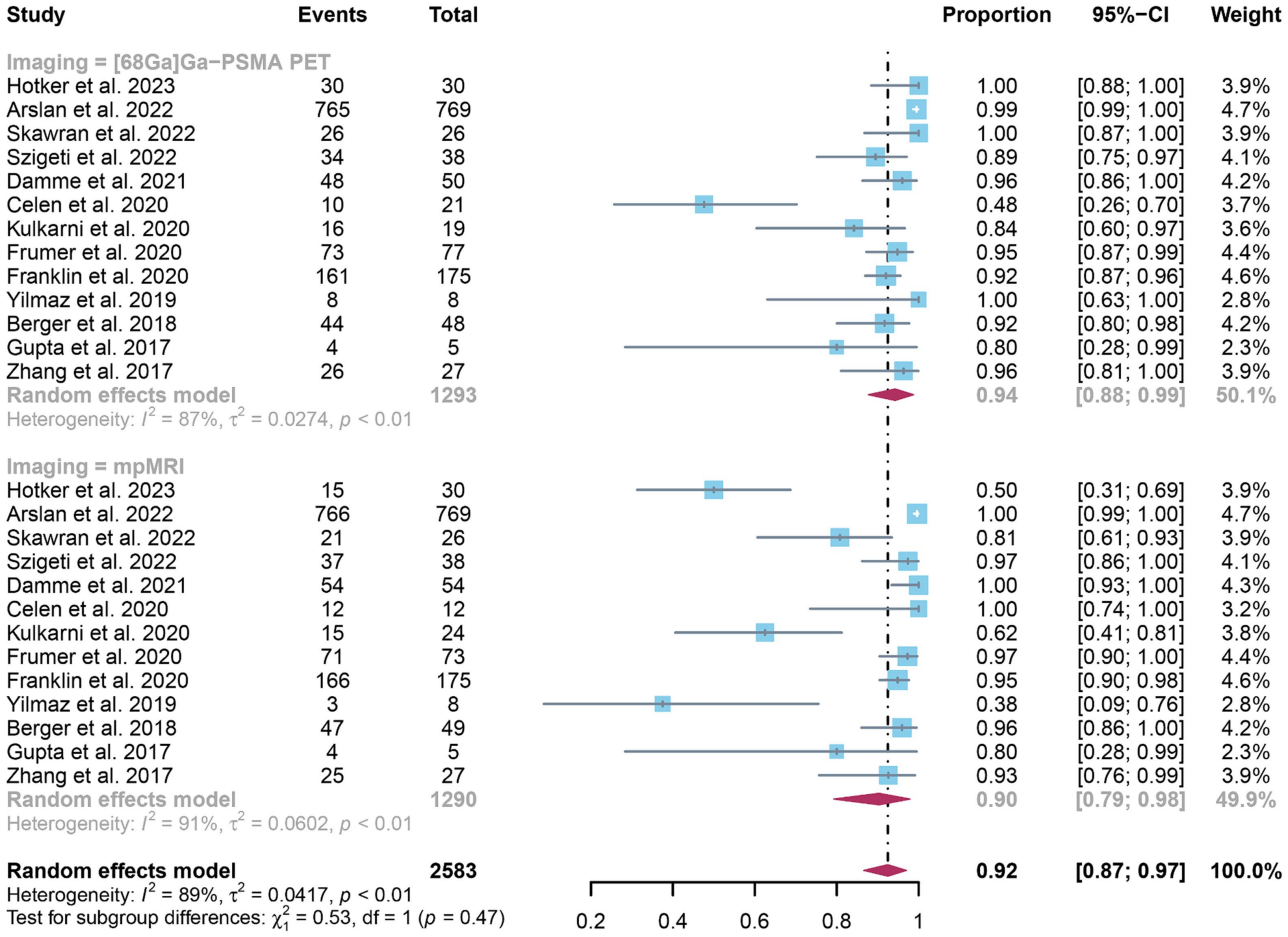
Forest plot showing the head-to-head comparison of pooled specificities for ^68^Ga-PSMA-11 PET and mpMRI in pelvic lymph node metastases of prostate cancer patients. The plot displays individual study estimates (squares) with corresponding 95% confidence intervals (horizontal lines) and the pooled specificity estimate (diamond) for both modalities. The size of the squares represents the relative weight of each study in the meta-analysis.

The analysis comprised 13 studies. The pooled specificity of ^68^Ga-PSMA-11 PET for diagnosing initial lymph node staging of prostate cancer was 0.94 (95% CI: 0.88–0.99), while mpMRI demonstrated an overall specificity of 0.90 (95% CI: 0.79–0.98), as illustrated in Figure 6. Notably, there was no significant difference in the overall specificity between ^68^Ga-PSMA-11 PET and mpMRI (*p* = 0.47), as shown in Figure 6.

**Figure 7:**
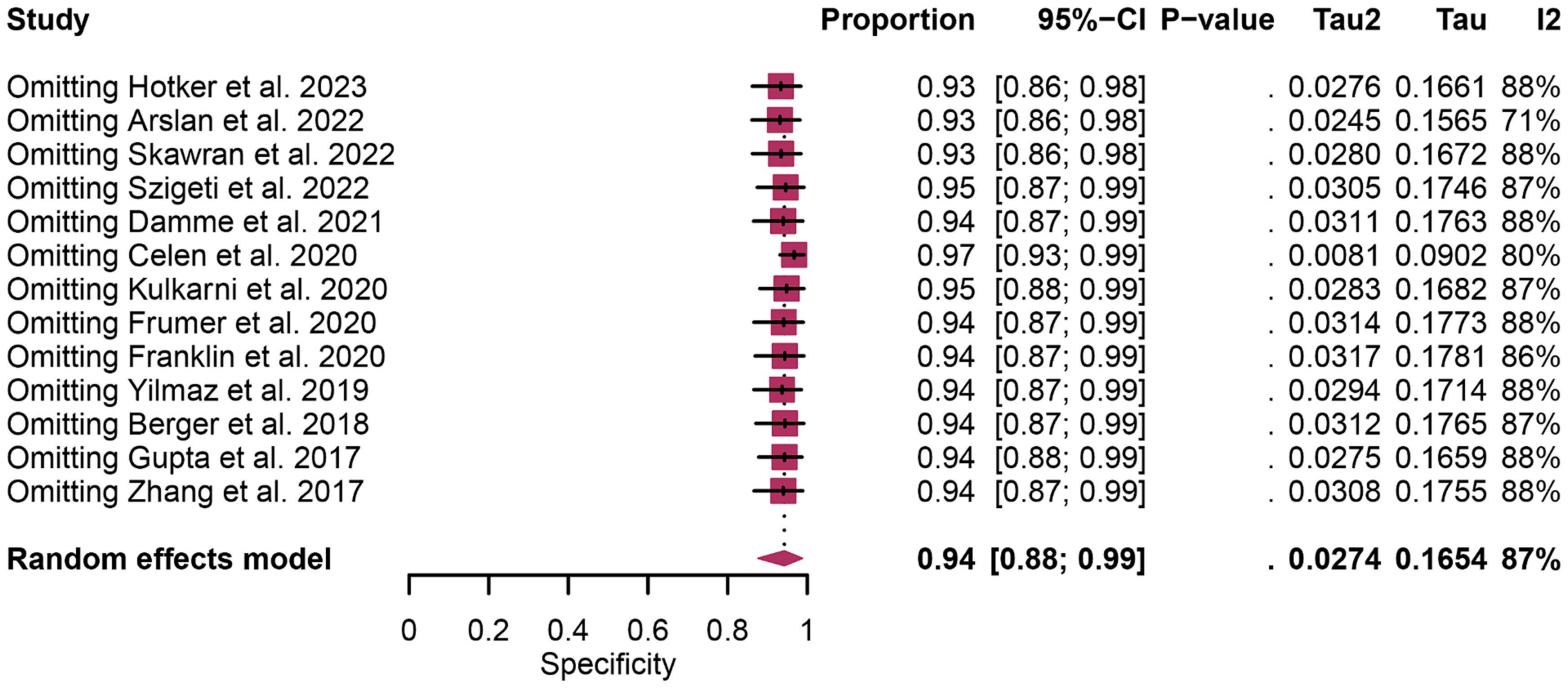
Forest plot showing the pooled specificity of ^68^Ga-PSMA-11 PET in pelvic lymph node metastases of prostate cancer patients. The plot displays individual study estimates (squares) with corresponding 95% confidence intervals (horizontal lines) and the pooled specificity estimate (diamond) for both modalities. The size of the squares represents the relative weight of each study in the meta-analysis.

**Figure 8:**
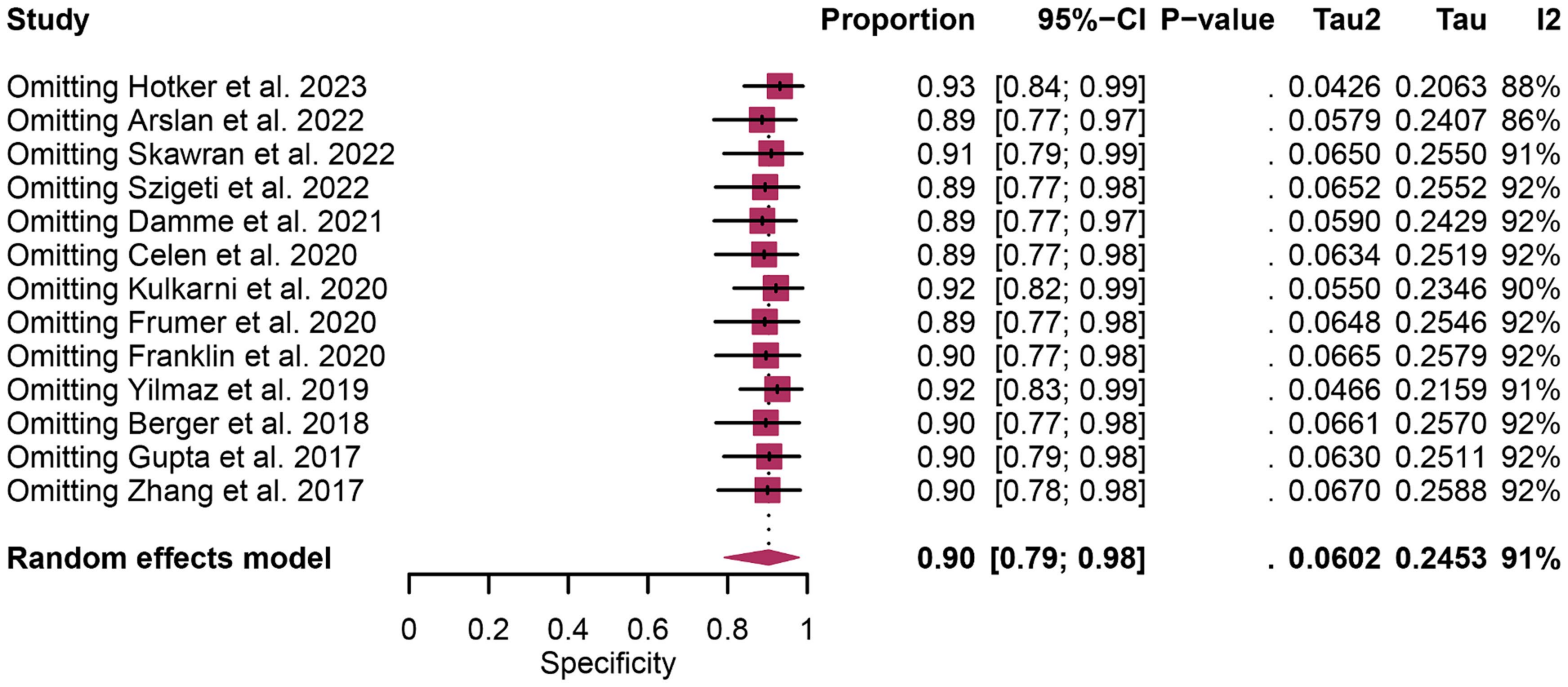
Forest plot showing the pooled specificity of mpMRI in pelvic lymph node metastases of prostate cancer patients. The plot displays individual study estimates (squares) with corresponding 95% confidence intervals (horizontal lines) and the pooled specificity estimate (diamond) for both modalities. The size of the squares represents the relative weight of each study in the meta-analysis.

In terms of heterogeneity, the pooled overall specificity exhibited *I*^2^ values of 87% for ^68^Ga-PSMA-11 PET and 91% for mpMRI, indicating substantial heterogeneity (Figures 7, 8). Interestingly, further analysis revealed that the number of patients included in the mpMRI analysis (>50 vs. ≤50, *p* = 0.02) may be the source of this heterogeneity, as detailed in Table 4. However, sensitivity analysis did not identify any potential sources of heterogeneity and the results remained stable, with only minor variations observed (ranging from 0.93 to 0.97 for ^68^Ga-PSMA-11 PET and from 0.89 to 0.93 for mpMRI), as depicted in Figures 7, 8.

**Table 4:** Meta-regression analysis for specificity of ^68^Ga-PSMA-11 PET and mpMRI.

Covariate	Studies	Specificity for ^68^Ga-PSMA-11 PET (95% CI)	*p*-value	Specificity for mpMRI (95%CI)	*p*-value
Region			0.38		0.51
Oceania	2	0.92 [0.88; 0.95]		0.95 [0.92; 0.98]	
Asia	7	0.91 [0.76; 1.00]		0.89 [0.70; 1.00]	
Europe	4	0.97 [0.92; 1.00]		0.88 [0.60; 1.00]	
Number of patients included			0.64		0.02
>50	6	0.95 [0.89; 0.99]		0.96 [0.87; 1.00]	
≤50	7	0.93 [0.78; 1.00]		0.82 [0.62; 0.96]	
Reference standard			0.62		0.53
PLND	8	0.93 [0.82; 0.99]		0.89 [0.75; 0.98]	
ePLND	4	0.96 [0.84; 1.00]		0.99 [0.94; 1.00]	
rPLND	1	1.00 [0.63; 1.00]		0.38 [0.09; 0.76]	
Image analysis			0.09		0.15
Visual and semiquantitative	12	0.93 [0.86; 0.98]		0.89 [0.77; 0.97]	
Visual	1	0.99 [0.99; 1.00]		1.00 [0.99; 1.00]	
Study design			0.69		0.49
Prospective	1	0.89 [0.75; 0.97]		0.97 [0.86; 1.00]	
Retrospective	12	0.95 [0.87; 0.99]		0.89 [0.77; 0.98]	

### 3.5 Publication bias of ^68^Ga-PSMA-11 PET and mpMRI for diagnosis of initial lymph node staging of prostate cancer

Funnel plot asymmetry test showed that there was a significant publication bias for specificity of ^68^Ga-PSMA-11 PET and mpMRI (Egger’s test: *p* = 0.01 and *p* = 0.00), and no significant publication bias was observed for sensitivity of ^68^Ga-PSMA-11 PET and mpMRI (Egger’s test: *p* = 0.89 and *p* = 0.41) (Supplementary Figures S1–S4).

## 4 Discussion

In the realm of diagnosing initial lymph node staging in prostate cancer, there exists considerable uncertainty and debate regarding the comparative diagnostic effectiveness of ^68^Ga-PSMA-11 PET and mpMRI. In 2022, Arslan et al. (20) stated that the sensitivity of both PSMA-PET/CT and mpMRI in the detection of metastatic lymph nodes was low. Berger et al. (19) highlighted the superior lesion detection capabilities of PSMA-PET/CT, particularly in terms of sensitivity, as compared to mpMRI. Conversely, Zhang et al. (8) found no significant difference in the detection of lymph node metastases (LNMs), particularly with respect to the diameter of the LNMs, between these two modalities. This discrepancy in findings underscores the ongoing debate and the need for comprehensive meta-analyses to clarify these differences. To mitigate the impact of bias and enhance the internal validity and reliability, this study using histopathology as the reference, presents a head-to-head comparison of ^68^Ga-PSMA-11 PET and mpMRI.

In our meta-analysis, the pooled sensitivity of ^68^Ga-PSMA-11 PET in the initial lymph node staging of prostate cancer was found to be 0.73 (95% CI: 0.51–0.91), while mpMRI demonstrated a sensitivity of 0.49 (95% CI: 0.30–0.68). It can be observed that ^68^Ga-PSMA-11 PET demonstrates higher sensitivity in detecting early lymph node metastasis in prostate cancer. However, no significant difference was observed. Wang et al. (22) evaluated the diagnostic performance of ^68^Ga-PSMA-11 PET in comparison to mpMRI for prostate cancer lymph node staging. Their article suggests that ^68^Ga-PSMA-11 PET/CT exhibits a higher sensitivity (71% vs. 40%). However, statistical tests for differences in sensitivity and specificity between the two diagnostic tools were not performed in their article. Our study conducted statistical tests for differences to determine if there are significant difference in performance between these two methods, rather than merely observing a trend.

Furthermore, the specificity was 0.94 (95% CI: 0.88–0.99) for ^68^Ga-PSMA-11 PET and 0.90 (95% CI: 0.79–0.98) for mpMRI in our study. Wang’s study suggested noting comparable specificity (92% vs. 92%) between the two methods. However, this contrasts with the findings of Chow et al. (23). The direct comparison in the study revealed a significant specificity advantage of 15.0 percentage points for PSMA-PET (95% CI 6.7–23.2; *p* < 0.001). This may be related to this study’s use of various tracers in PSMA-PET imaging, such as ^68^Ga-PSMA-11, 18F-DCFPyL, 18F-PSMA-1007, leading to higher heterogeneity in the articles. In cases of high heterogeneity, their study did not employ techniques such as meta-regression or sensitivity analysis to explore the source of heterogeneity. Therefore, in our study, we conducted meta-regression and sensitivity analysis specifically for one imaging tracer, ^68^Ga-PSMA-11, and incorporated the latest studies.

In addition, our study find substantial heterogeneity was identified in the sensitivity and specificity of ^68^Ga-PSMA-11 PET and mpMRI, as evidenced by high I^2^ values (84 and 79% for sensitivity, 87 and 91% for specificity, respectively). Hence, in response to this high level of heterogeneity, we utilized meta-regression and sensitivity analysis to explore potential sources of this heterogeneity. Through meta-regression analysis, only patient numbers emerged as a statistically significant factor for the specificity of mpMRI (*p* < 0.05). This finding suggests the influence of sample size on heterogeneity, yet the limited identification of sources indicates the complexity of factors affecting diagnostic tool performance. The complexity of this outcome may stem from multiple factors. Firstly, the diversity in study samples, encompassing variations in geography, ethnicity, and age among populations, might not have been fully addressed in the analysis. Secondly, the differences in the application of diagnostic tool models and the subjective assessment criteria across various studies may obscure the true sources of heterogeneity. Additionally, although factors such as region, number of patients included, reference standard, image analysis, and study design were incorporated in the meta-analysis, pinpointing specific sources of heterogeneity remains a formidable challenge.

The current meta-analysis indicates that ^68^Ga-PSMA-11 PET exhibits similar sensitivity to mpMRI in detecting initial lymph node staging in prostate cancer patients. However, it’s crucial to consider the availability of ^68^Ga-PSMA-11 PET, which may not be uniformly accessible across medical centers and can be influenced by location and resources. Moreover, one of the main limitations of ^68^Ga-PSMA-11 PET is the potential exposure to ionizing radiation, particularly concerning for younger patients or those requiring repeated imaging exams. On the other hand, mpMRI combines multiple imaging modalities, providing detailed anatomical and functional information about tumors. Its lower economic cost compared to ^68^Ga-PSMA-11 PET contributes to its widespread use in clinical practice. The choice between the two modalities will depend on factors such as the clinical scenario, accessibility of the imaging technique, and physician preferences.

Our study has several limitations that need to be acknowledged. Firstly, despite employing rigorous statistical methods, we were unable to identify specific sources of heterogeneity for sensitivity and specificity. This suggests that there may be underlying complexities, highlighting the need for further research to pinpoint these sources of heterogeneity. Secondly, due to the limited number of included studies, we were unable to divide our analysis into patient-based and lesion-based analyses. Future head-to-head comparison studies focusing on these distinct analysis methods may provide more precise and accurate conclusions. Thirdly, it’s important to note that the majority of studies included in our meta-analysis were retrospective (only one out of 13 was prospective), which could potentially introduce bias into our findings.

## 5 Conclusion

Our findings indicated that ^68^Ga-PSMA-11 PET and mpMRI exhibit similar sensitivity and specificity in the diagnosis of initial lymph node staging of prostate cancer. However, given that most included studies were retrospective, further prospective studies with larger sample sizes are essential to validate these results.

## Data availability statement

The original contributions presented in the study are included in the article/Supplementary material, further inquiries can be directed to the corresponding author.

## Author contributions

YW: Data curation, Formal analysis, Methodology, Software, Writing – original draft. RJ: Data curation, Software, Writing – original draft. HW: Formal analysis, Methodology, Writing – original draft. QZ: Conceptualization, Supervision, Validation, Visualization, Writing – review & editing.
