# Supplementary material for: 68Ga-PSMA-11 PET and mpMRI in the diagnosis of initial lymph node staging of prostate cancer: a head-to-head comparative meta-analysis
Source: Front Med (Lausanne). 2024 Jun 20;11:1425134. doi: 10.3389/fmed.2024.1425134 (PMC11222328; doi:10.3389/fmed.2024.1425134)

Supplementary Table 1. Search strategy in PubMed and Embase

| Database | Search strategy |
| --- | --- |
| PubMed | ((("Positron-Emission Tomography"[Mesh]) OR ((((((Positron Emission Tomography) OR (Positron-Emission Tomography Imaging)) OR (Tomography Imaging, Positron-Emission)) OR (Tomography, Positron-Emission)) OR (PET Scan)) OR (PET Imaging))) AND (("Multiparametric Magnetic Resonance Imaging"[Mesh]) OR (((((mpMRI) OR (Multiparametric MRI)) OR (MRIs, Multiparametric)) OR (Multiparametric MRIs)) OR (mp-MRI)))) AND (("Prostatic Neoplasms"[Mesh]) OR ((((((((Prostate Neoplasms) OR (Neoplasm, Prostate)) OR (Neoplasm, Prostatic)) OR (Prostate Cancer)) OR (Cancer of the Prostate)) OR (Prostatic Cancer)) OR (Prostatic Cancers)) OR (Cancer of Prostate))) |
| Embase | ('positron emission tomography'/exp OR ‘Positron Emission Tomography’:ab,ti OR ‘Positron-Emission Tomography Imaging’:ab,ti OR ‘Tomography Imaging, Positron-Emission’:ab,ti OR ‘Tomography, Positron-Emission’:ab,ti OR ‘PET Scan’:ab,ti OR ‘PET Imaging’:ab,ti) AND ('multiparametric magnetic resonance imaging'/exp OR 'mpMRI':ab,ti OR 'Multiparametric MRI':ab,ti OR 'MRIs, Multiparametric':ab,ti OR 'Multiparametric MRIs':ab,ti OR ‘mp-MRI’:ab,ti) AND ('prostate tumor'/exp OR 'Prostate Neoplasms':ab,ti OR 'Neoplasm, Prostate':ab,ti OR 'Neoplasm, Prostatic':ab,ti OR 'Prostate Cancer':ab,ti OR 'Cancer of the Prostate':ab,ti OR 'Prostatic Cancer':ab,ti OR 'Prostatic Cancers':ab,ti OR 'Cancer of Prostate':ab,ti) |

Supplementary Figure 1. Sensitivity funnel plot of ^68^Ga-PSMA-11 PET


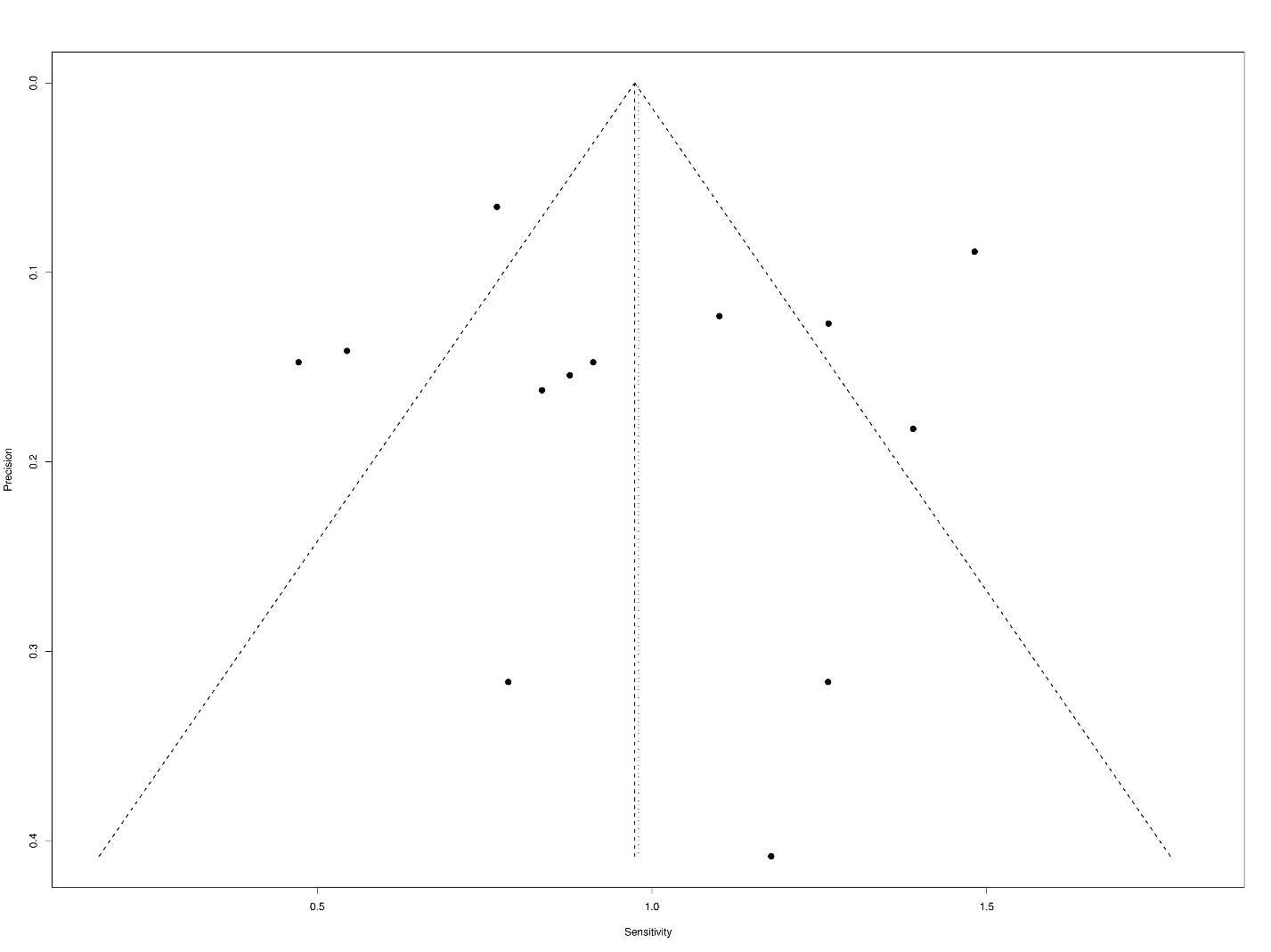


Supplementary Figure 2. Specificity funnel plot of ^68^Ga-PSMA-11 PET


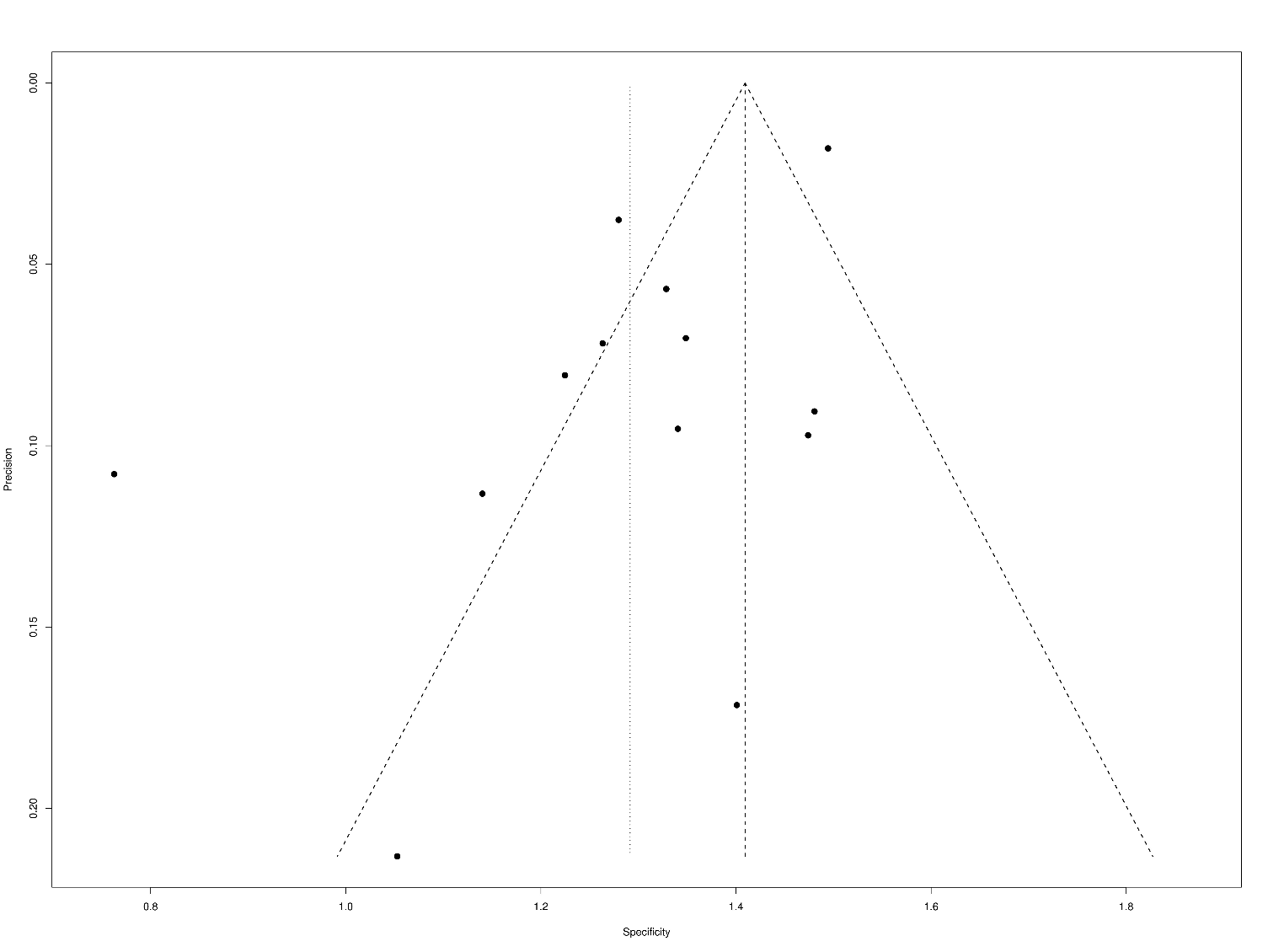


Supplementary Figure 3. Sensitivity funnel plot of mpMRI


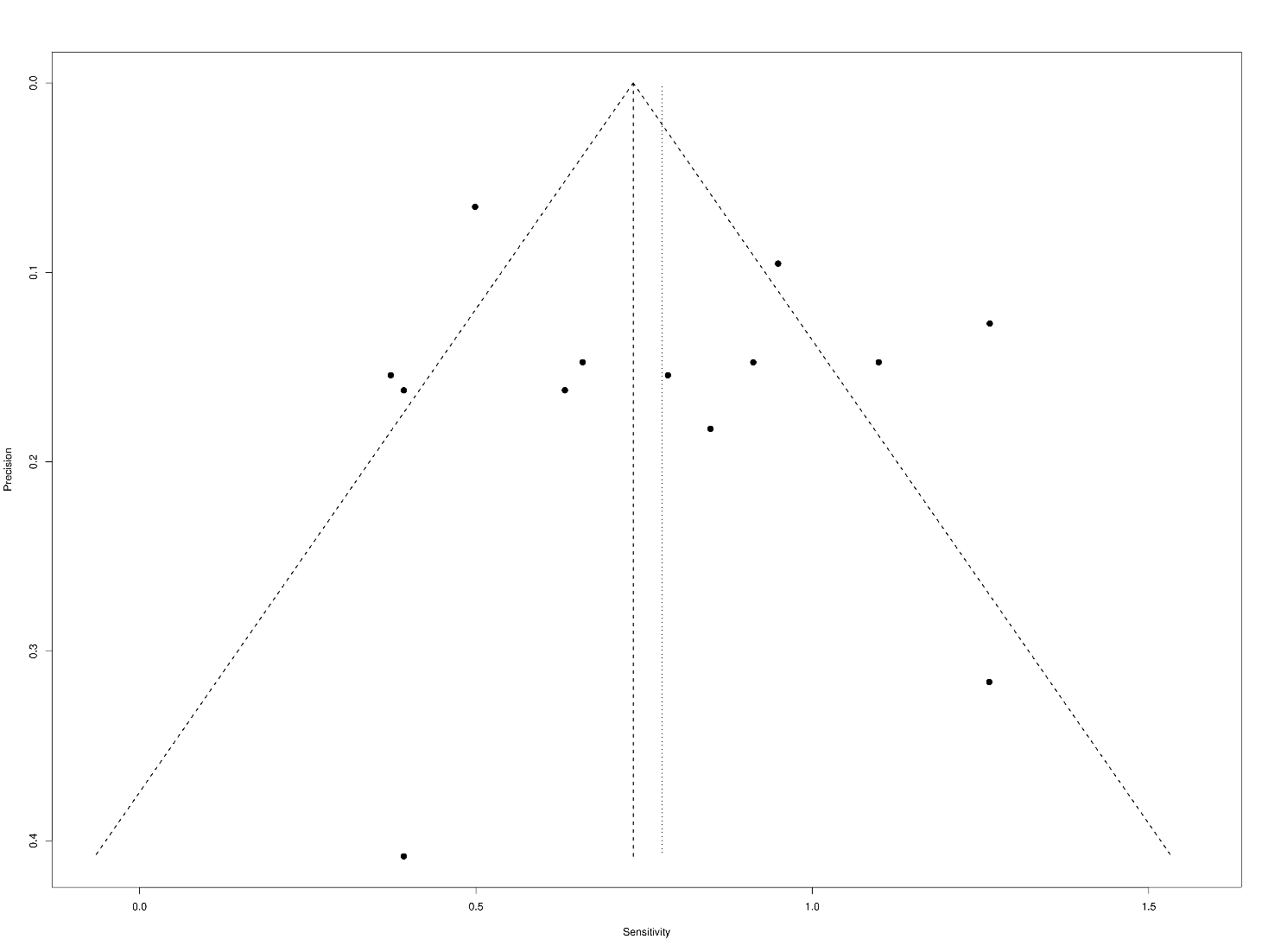


Supplementary Figure 4. Specificity funnel plot of mpMRI


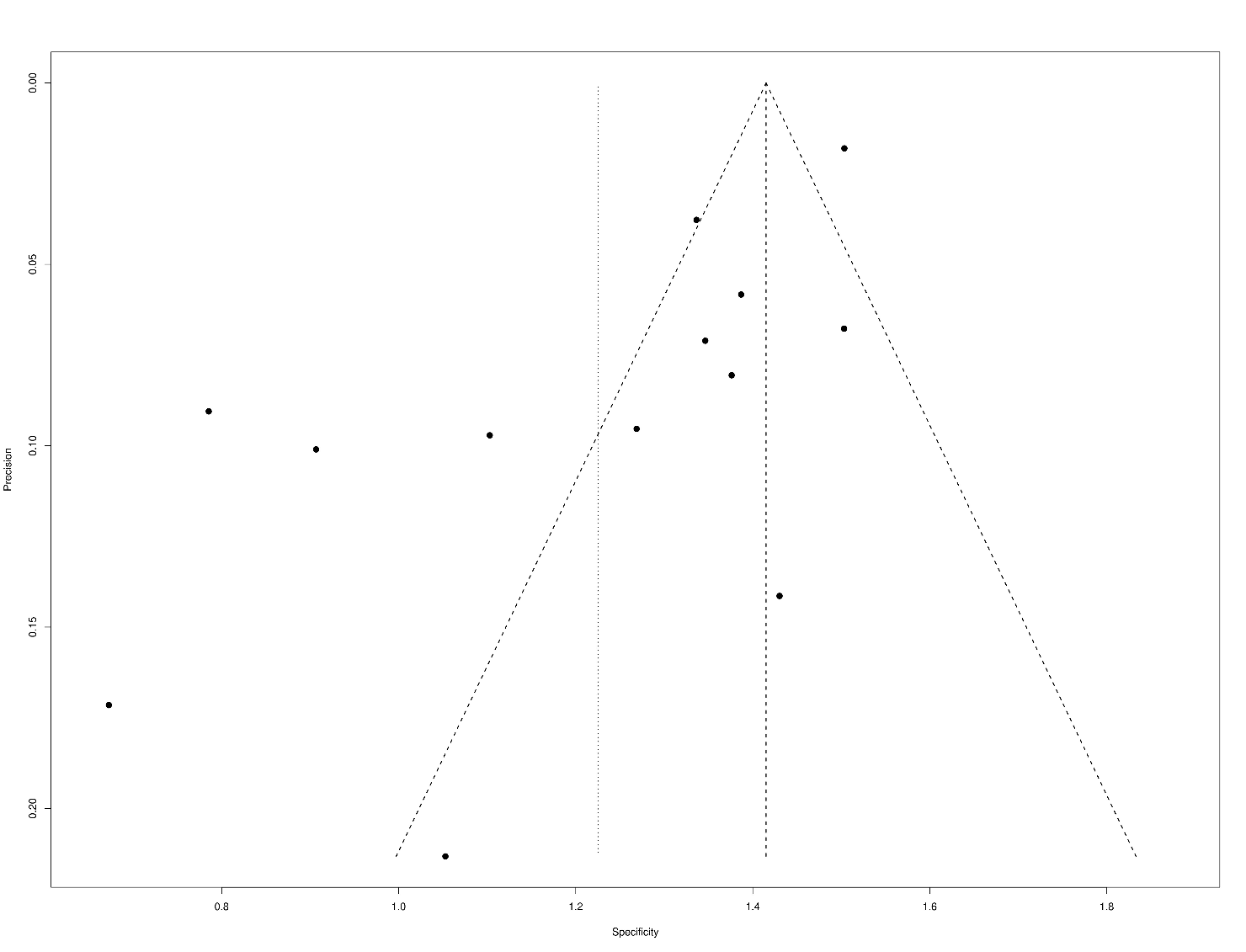

Supplement: Supplementary file 1 [file Data_Sheet_1.docx]
